# Obesity and hypertension in an Iranian cohort study; Iranian women experience higher rates of obesity and hypertension than American women

**DOI:** 10.1186/1471-2458-6-158

**Published:** 2006-06-20

**Authors:** Hossein Bahrami, Mohsen Sadatsafavi, Akram Pourshams, Farin Kamangar, Mehdi Nouraei, Shahriar Semnani, Paul Brennan, Paolo Boffetta, Reza Malekzadeh

**Affiliations:** 1 Digestive Disease Research Center (DDRC), Tehran University of Medical Sciences, Tehran, Iran; 2 Departments of Epidemiology and Internal Medicine, Johns Hopkins Schools of Public Health and Medicine, Baltimore, MD, USA; 3 Department of Health Care and Epidemiology, University of British Columbia, Vancouver, BC, Canada; 4 National Cancer Institute (NCI), National Institute of Health (NIH), Bethesda, USA; 5 Department of Internal Medicine, Golestan University of Medical Sciences, Gorgan, Iran; 6 International Agency for Research on Cancer, Lyon, France

## Abstract

**Background:**

Once considered as the main public health problem in developed countries, obesity has become a major problem throughout the world and developing countries, like Iran, are joining the global obesity pandemic. We determined the prevalence of overweight, obesity, and hypertension in a large cohort of Iranians and compared age-adjusted rates with the rates in the US.

**Methods:**

Golestan Cohort Study is a population-based study of 8,998 men and women, aged 35-81 years, from urban and rural areas. Anthropometric parameters were measured by interviewers. Prevalence rates were directly adjusted to the 2000 United States standard population.

**Results:**

The age-adjusted prevalence rates of overweight (BMI ≥ 25 kg/m2) and obesity (BMI ≥ 30 kg/m2) in this Iranian population were 62.2% and 28.0%, respectively.  Both overweight and obesity were more common in women than men. Age-adjusted prevalence of overweight was significantly higher in Iranian women compared to the American women (68.6% vs. 61.6%), while the age-adjusted prevalence of obesity is closer in these two populations (34.9% vs. 33.2%). Iranian men—compared to American men—had significantly lower age-adjusted prevalence of overweight (53.7% vs. 68.8%) and obesity (16.2% vs. 27.5%). Age-adjusted prevalence of hypertension was higher in Iranian women than American women (35.7% vs. 30.5%). Diabetes mellitus was reported in 6.2% of participants. Mean waist-to-hip ratio (WHR) among women was 0.96. Smoking rates in men and women were 33.2% and 2.2%, respectively.

**Conclusion:**

The prevalence of obesity, overweight, and hypertension in Iran is as high as the US. However, Iranian women are more obese than American women and Iranian men are less obese than their American counterparts. This discrepancy might be due to the low rate of smoking among Iranian women. Iranian women have higher mean WHR than what WHO has defined in 19 other populations.

## Background

Listing medical and health-related issues in the Middle East, the first things that come to one's minds might be problems like infectious diseases that are more common in developing countries. However, by merely focusing on specific types of endemic diseases in the region, we undermine significant problems like obesity and metabolic syndrome that might have equal or even greater impact on the health of people in these countries. Although these health issues are more discussed in developed countries, we should notice that most Middle Eastern countries are joining the global obesity pandemic and the problem becomes more significant when we consider the trend in developing countries toward the Western lifestyles. This westernization can potentially put the population of these countries at higher risk of obesity and metabolic syndrome than western countries. It is suggested that fetal growth and maternal nutrition have crucial effects on epigenetic programming of obesity, metabolic syndrome, diabetes, and hypertension [1-3] and in these countries we are dealing with a generation born in more traditional environment–and therefore more susceptible to obesity and insulin resistance–who is exposed to high-calorie foods and sedentary lifestyle. Failure of the scientific community in recognizing the problem will result in a Middle East with an alarming situation like what we see in Pima Indians in the US [4,5] or adults with prenatal exposure to Dutch famine in Europe [6,7]. As Horton has highlighted, we have an unusual opportunity to act in order to prevent the needless deaths of millions by focusing on risk factors of chronic diseases in low-income countries [8].

Obesity is a major medical and public health problem world-wide and by consequences like type 2 diabetes mellitus and insulin resistance ultimately causes several health conditions like cardiovascular and cerebrovascular diseases, certain types of cancers, hypertension, gallbladder disease, nonalcoholic steatohepatitis, and obesity-hypoventilation syndrome. In addition to overall obesity, abdominal obesity is considered as independent predictor of several risk factors and morbidity [9,10].

This paper estimates the prevalence of overweight, obesity, and abdominal obesity in Iran using the da large cohort study, the Golestan Cohort Study. The age-adjusted and age-specific prevalence rates will be compared to the rates in the United States. Considering the association of obesity with metabolic syndrome, we estimate the prevalence of hypertension and diabetes mellitus–two other criteria for metabolic syndrome and two independent risk factors for many comorbid conditions–in addition to obesity.

## Methods

### Study population

This study analyzes the data from the Golestan Cohort Study, a large study that is conducted in North-East of Iran. The study is an ongoing population-based cohort with the main objective of investigating the incidence of esophageal cancer and the associated risk factors. For this analysis, we included data from 8,999 participants recruited between 2002 and 2005. The study is sponsored by Digestive Diseases Research Center (DDRC) at Tehran University of Medical Sciences (Tehran, Iran), International Agency for Research on Cancer (IARC; Lyon, France), and Golestan University of Medical Sciences, Iran. One participant was excluded from the analysis because his height was missing. The study adhered to the Declaration of Helsinki [11] and was approved by the Institutional Review Board of the DDRC.

Residents were selected by household sampling and contacted at their home by expert local health professionals, who thoroughly explained them the purpose and procedure of the study. Individuals were invited to participate by attending the health service centers located in their area or the Golestan Cohort Study Center for interview. More than 75% of rural subjects and 62% of urban subjects who were invited to the study participated. Interviewers were local physicians and, in the case of dietary interviews, nutritionists who were trained for this purpose. Exclusion criteria were: (1) being a temporary resident; and (2) previous history of upper gastrointestinal cancer.

### Data collection

Before interview, a written informed consent was obtained from all study participants. Detailed data concerning alcohol consumption and smoking as well as type, duration, and estimated amount of drinking/smoking were collected. Demographic and anthropometric data were collected on all participants. Weight (kg), height (cm), waist and hip circumferences (cm) were measured by interviewers and recorded with precision of one unit. The values for weight were rounded to the nearest 0.5 kg and height was rounded to the nearest 0.1 cm. Subjects were wearing light clothes. Three anthropometric indices that were analyzed in this study were body mass index (BMI), waist-to-hip ratio (WHR), and waist circumference (WC). BMI was calculated using the formula weight(kg)/[height(m)]^2 ^and WHR was defined as the ratio of waist circumference (WC) to hip circumference. BMI is recognized as the measure of overall obesity. A BMI ≥ 25 kg/m^2 ^was defined as overweight and a BMI ≥ 30 kg/m^2 ^was defined as obesity. Individuals with BMI<18.5 kg/m^2 ^were considered as underweight. WHR and WC were used as measures of abdominal obesity. A WHR ≥ 0.95 in men and = 0.85 in women were considered as high WHR (abdominal obesity) [10]. Also, a WC>102 cm in men and >88 cm in women were considered as high WC [9].

Two recordings of systolic and diastolic blood pressures were obtained for each arm in sitting position. Hypertension was defined as any of these conditions: (1) average systolic blood pressure ≥ 140 mmHg; (2) average diastolic blood pressure ≥ 90 mmHg; (3) being known case of hypertension (diagnosed by a physician) or receiving medications for hypertension.

Diabetes mellitus was defined by self-report. A questionnaire including questions about different mild, moderate, and strenuous physical activities were filled for all participants. Metabolic equivalent (MET) for various activities was calculated based on an updated Compendium of Physical Activities [12]. Data on calorie intake calculated from food-frequency questionnaire (FFQ) was available for 4,048 participants. Total daily calorie intake was calculated using a Food Processor^© ^Nutrition Analysis Software (Elizabeth Stuart Hands and Associates (ESHA) Research, Salem, Oregon).

### Statistical analysis

The data are presented as crude and adjusted prevalence rates, standard errors, and 95% confidence intervals (binomial exact confidence intervals). Prevalence rates have been directly adjusted to the 2000 United States standard population [13] using 5-year age groups. The main reason for this adjustment was to compare the results with the results of the Third National Health and Nutrition Examination Survey (NHANES III) in the United States [14]. Since the age range in our study population was from 35 to 81 years, we considered one 81-year-old and two 80-year-old participants in the age group 75–79.

Generalized linear regression model is used to estimate predictors of BMI. We used logistic regression to determine the association of binary outcomes with independent covariates. Model assumptions and robustness of our results to highly influential observations were checked (using regression diagnostics, dbetas, and dfits). Data on continuous variables are presented as mean ± standard deviation (SD). Prevalence rates are presented as percent and 95% confidence interval (CI). T test and chi-square test have been used to compare numeric and categorical variables, respectively. Data analyses were performed using Stata^® ^statistical software (Version 8.0, Stata Inc., College Station, TX).

## Results

### Prevalence of obesity and overweight

Excluding one individual whose height was missing from the analysis, the study population consisted of 8,998 participants (3,786 men and 5,212 women) with mean ± SD age of 52.8 ± 9.4 years (range 35 to 81 years). Among these participants, 5,182 individuals (57.6%) were from urban population and 3,817 individuals (42.4%) were from rural area. Table 1 summarizes the general characteristics of the study population.

The overall prevalence of overweight (BMI ≥ 25 kg/m^2^) was 63.5% (95% CI: 62.5%–64.5%). The prevalence of overweight was slightly higher in younger age groups, but it was significantly reduced after 65 years old (p-value for trend < 0.01). The prevalence of overweight was significantly (p-value < 0.01) higher in women than men (69.7% vs. 54.9%).

The overall prevalence of obesity (BMI ≥ 30 kg/m^2^) was 28.4% (95% CI: 27.4%–29.3%). The pattern of change in the prevalence of obesity across different age groups was similar to the prevalence of overweight. Women had a significantly higher prevalence of obesity compared to men (36.7% vs. 17.0%, p-value < 0.01).

**Figure 1:**
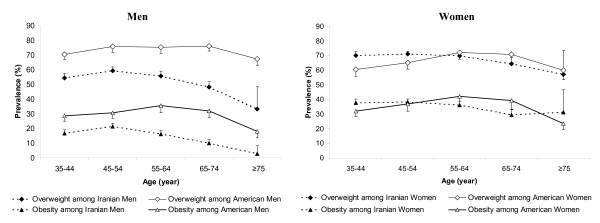
Age-specific prevalence of obesity and overweight among Iranian and American participants of the Golestan Cohort Study and Third National Health and Nutrition Examination Survey (NHANES III), respectively. Note: Error bars indicate 1.96*standard error.

Table 2 compares the unadjusted and adjusted prevalence rates of obesity and overweight in this Iranian population to the rates in the United States. Adjustments have been performed in both populations to the 2000 US standard population using 5-year age groups. The adjusted prevalence rates of overweight and obesity in this Iranian population were 62.2% and 28.0%, respectively. These rates are very close to similar rates in the US population (65.1% and 30.4%, respectively). However, gender-specific rates demonstrate that the age-adjusted prevalence of overweight is significantly higher in Iranian women compared to the American women (68.6% vs. 61.6%), while the age-adjusted prevalence of obesity is closer in these two populations (34.9% vs. 33.2%). On the contrary, Iranian men have significantly lower age-adjusted prevalence of overweight (53.7% vs. 68.8%) and obesity (16.2% vs. 27.5%). Figure 1 illustrates the gender- and age-specific prevalence rates of obesity and overweight in these two populations. As illustrated in this figure, Iranian men have higher prevalence of both obesity and overweight in all age-groups shown in this figure, while Iranian women have higher prevalence of obesity and overweight in lower age-groups, which constitute considerable proportions of the population (about 62% of the US population above 35 years old in 2000 were in the age-groups 35 to 55, where Iranian women have a significantly higher prevalence of obesity).

In the logistic regression model for both obesity and overweight, the significant difference between genders did not change considerably after adjustment for age, education, and smoking. The variables that were associated with lower odds of obesity in the logistic regression model were older ages, male gender, living in rural areas, lower level of education, and smoking. Odds ratio (OR) of being obese comparing females to males was 1.8 (95% CI: 1.6–2.0). Ethnicity, alcohol consumption, and physical activity were not significant predictors of the obesity. Similar results were obtained in the logistic regression model for overweight. OR of being overweight comparing women to men was 2.7 (95% CI: 2.4–3.1). The only difference was that alcohol consumption was associated with higher odds of being overweight. Linear regression model using BMI as the outcome showed similar results, although the association of alcohol consumption showed a week association with higher BMI (p-value: 0.095).

Overall prevalence rates of overweight among men and women in rural areas were 50.1% and 64.6%, respectively, while these rates in urban areas were 58.4% and 73.5%, respectively. The prevalence rates of obesity among men and women living in rural areas were 14.2% and 33.6%, respectively, which were lower than the rates in urban areas, 18.9% and 38.9%, respectively. There prevalence of obesity and the factors associated with it were similar in different ethnic groups.

High WHR was observed in 74.5% of the study population (95% CI: 73.6%–75.4%). The prevalence rates of high WHR were 54.7% (95% CI: 53.1%–56.3%) among men and 88.9% (95% CI: 88.0%–89.7%) among women. Since WHR is suggested to be independent risk factor of cardiovascular diseases, we looked at the overlap between high WHR and high BMI. The overall percent agreement and kappa statistic between high WHR and overweight were 76.7% and 0.44 (standard error: 0.01), respectively. About 49.8% of subjects with normal weight (18.5 ≤ BMI<25 kg/m^2^) had high WHR and 9.7% of overweight participants had low WHR. The agreement between high WHR and overweight was higher in men than in women (kappa statistics: 0.58 and 0.27, respectively). In other words, women have higher chance of having high WHR even if they have normal weight. The other finding was that 77.3 % of women with normal BMI had high WHR, while only 4.3% of women with low WHR had high BMI. These proportions were 24.8% and 19.1%, respectively, among men. The similarity between these two proportions among men brings about the rather similar prevalence rates of overweight and high WHR among men, while prevalence of high WHR is significantly higher than the prevalence of overweight among women.

About 80.7% of participants had either high BMI ≥ 25 kg/m^2 ^or high WHR (95% CI: 79.8%–81.5%). This rate was 65.2% (95% CI: 63.7%–66.7%) among men and 91.9% (91.1%–92.6%) among women. High waist circumference (more than 102 cm in men and 88 cm in women) was observed in 58.4% of participants.

The aforementioned analyses were repeated in a subset of the study population (4,408 consecutive participants who have been recruited in the last half of the study) for whom FFQ were filled and therefore data on daily calorie intake were available. This subset of the population consisted of 1,731 males and 2,317 females with a mean age of 53.1 ± 9.4 years. All these participants were recruited during second and third years of accrual. There was no association between gender and daily calorie intake. In the logistic regression model, participants in the highest tertile of daily calorie intake had higher prevalence of overweight compared to the participants in lower two tertiles (OR: 1.15, 95% CI: 1.00–1.33, p-value: 0.04). However, daily calorie intake was not a significant predictor of obesity, abdominal obesity (estimated by high WHR), and BMI in the multivariate logistic and linear regression models.

### Prevalence of hypertension and diabetes mellitus

Unadjusted Prevalence of hypertension was 32.5% (95% CI: 31.6%–33.5%) and hypertension was more common in women than men (OR: 1.2, 95% CI: 1.1–1.4). Table 3 compares the crude and adjusted prevalence of hypertension in our study with the results of NHANES III. While unadjusted prevalence rate of hypertension in Iranian and American women are rather similar, adjusted prevalence rate of hypertension in Iranian women is significantly higher than Americans. Mean BMI in participants with and without hypertension was 27.6 and 27.3 kg/m^2 ^(p-value: 0.08). In logistic regression model, diabetes mellitus and female gender were the only risk factors associated with hypertension. There was no significant association between hypertension and body weight.

Diabetes mellitus was reported in 6.1% (95% CI: 5.7%–6.7%) of participants. In logistic regression model, hypertension and living in urban areas were only predictors of diagnosed diabetes mellitus. Prevalence of diabetes mellitus in men and women was 4.4% and 7.4%, respectively. In multivariable logistic regression model, odds of having diagnosed diabetes mellitus in women was higher than men (OR: 1.9; 95% CI: 1.5–2.3). Diabetic participants had significantly higher BMI (p-value: 0.04).

### Alcohol and smoking

Alcohol consumption rate was 7.5% (95% CI: 7.0%–8.1%) and men had a significantly higher rates of alcohol consumption comparing to women (17.8% and 0.1%, respectively, p-value < 0.01).

Current smoking rates were 33.2% (95% CI: 32.4%–34.0%) in men and 2.2% (95% CI: 2.0%–2.4%) in women. Ever smoking rates in men and women were 47.0% and 2.6%, respectively. Adjusting for smoking in multivariable logistic models for obesity and overweight reduced the magnitude of the differences between genders.

## Discussion

Once considered as the main public health problem of the developed countries, obesity has become a major problem throughout the world and the prevalence of obesity is increasing in most developing countries as well.

The first important finding of our study was that the estimated overall prevalence of obesity in an Iranian population is quite comparable to the US [14] and higher than United Kingdom, France, Netherlands, and Italy [15-19]. These results are consistent with similar reports from Iran [20-22] as well as other countries in Middle East [23-30]. Even some reports from some Middle Eastern countries show a higher prevalence of obesity comparing to the US [31]. While World Health Organization (WHO) has estimated that the mean BMI in Africa and Asia is about 22–23 Kg/m^2 ^[32], the average BMI in our study as well as in other studies in Middle East is similar to the North American and European countries. The increasing prevalence of obesity has brought about the increase in myriad of public health consequences in these countries. Currently, cardiovascular diseases (47%) and cancer (14%) account for more than 60% of mortality in Iran [21].

One major concern in comparing our results with the results of the NHANES III was the generalizability of these findings to the total population in Iran. Our study was a large population-based study and consisted of both urban and rural populations. However, the study population consisted of individuals older than 35 years old. In order to address this limitation, we compared the age-specific rates between Iranian and American populations. Also, our study population had higher proportion of females comparing to the total population of Iran. This might have resulted in a slight overestimation of the prevalence of obesity in Iranian general population. However, even if we calculate the prevalence rate based on the male/female ratio in the total population of Iran (approximately 1:1) [33], the adjusted prevalence rate of overweight and obesity will be 61.2% and 25.6%, respectively, which are still comparable to the rates in the United States. In addition, our results were consistent with the results of Tehran Lipid and Glucose Study (TLGS), another large cohort study that was carried out in urban population of the capital city of Tehran [21]. In TGLS, 63.1% of the adult population over 20 years old were overweight (BMI ≥ 25 kg/m^2^) and 23.1% of them were obese (Table 3). This consistency in findings indicates a reasonable generalizability of our results, although generalizability is still one of the limitations of this study.

Since the standardized prevalence rates in the US population are calculated using all age-groups, one of our concerns was if the adjusted rates in US are affected by the lower prevalence of obesity in lower age-groups. Therefore, we calculated the truncated age-adjusted prevalence of obesity and overweight in both populations (using the population above 35 years old). Because the age-specific rates from NHANES III were available in 10-year intervals, these rates are calculated using 10-year age intervals and therefore, the results are slightly different from what reported above. The truncated adjusted prevalence of overweight in our study and in NHANES III was 53.7% and 66.7%, respectively, among men and 68.8% and 57.3%, respectively, among women. Truncated adjusted prevalence rates of obesity in Iranian men and women were 16.1% and 36.2%, respectively, and these rates were 23.2% and 28.8% in American population.

The other important result of this study was the discrepancy in gender distribution of obesity and overweight in Iran comparing to the US. This discrepancy was also observed in several other studies conducted in the Middle East [21,23,25,29], although some studies have shown a pattern close to what is observed in the United States [28]. As it is shown in Table 3, the results of TGLS were completely consistent with our results. In a study by Yumuk et al [23], the prevalence of obesity among Turkish women and men was 32.4% and 14.1%, respectively, while the prevalence of overweight among men and women was 65.9% and 50.4%, respectively. In Saudi Arabia, the prevalence of obesity is estimated to be between 17–44% in women and 13–26% in men [25-27,34] and in Egypt, the prevalence of obesity ranges from 40.6% among women living in urban areas to 6% among men living in rural areas [29].

Therefore, although the overall prevalence of overweight and obesity in Iranian population–as well as many other Middle Eastern populations–is rather similar to the American population, Iranian women tend to be more obese than American women and Iranian men are less obese than their American counterparts. As explained above, both our study and the TLGS demonstrated a significantly higher prevalence of overweight and obesity among women. We tested several hypotheses that could potentially explain this difference. One hypothesis was that probably these results are explained by differences in physical activity or calorie intake. In other words, Iranian women might have less physical activity and/or higher calorie intake comparing to Iranian men and American women. However, neither physical activity nor calorie intake were significantly different between Iranian men and women. Lack of association between gender and physical activity or calorie intake reduces the probability that the gender discrepancy in prevalence of obesity is due to these factors. Since physical activity was estimated using a different questionnaire, the direct comparison between our results and the results of studies conducted in developed countries was not possible. Although the total calorie intake was not associated with gender and therefore has low probability of being a confounder, it is possible that differences in the dietary contents–rather than total calorie intake–account for part of the discrepancy between Iranian men and women as well as the discrepancy between Iranian people and their American counterparts. Another hypothesis is the constitutional and biological differences between two populations or residual confounding due to the factors that we have not measured in this study.

The strongest hypothesis that was supported by this study is the difference in smoking habits. Smoking is shown to be associated with lower BMI and even some authors have suggested that decreasing rates of smoking in the US is one of the important contributing factors to the rising prevalence of obesity in the US [35]. Current smoking rates among men and women in our study were 33.2% and 2.2%, respectively, while these rates among American men and women are 24.8% and 20.1%, respectively. Therefore differences in smoking rates is consistent with discrepancy between Iranian men and women as well as the discrepancy between Iranian and American population.

Other important results of our study were the prevalence rates of hypertension and diabetes mellitus as two other risk factor of cardiovascular diseases and two other criteria of metabolic syndrome. Prevalence of hypertension in our study was quite comparable with prevalence rates in the US. Diabetes mellitus was measured based on self-report and since a significant proportion of diabetic cases are not clinical, our results significantly underestimates the prevalence of diabetes. Consequently, the interpretation of our findings should be limited to the diagnosed cases of diabetes. Prevalence of diabetes in this Iranian population was slightly lower than the US population–with about 8% prevalence of diabetes–and is higher than what is reported in some neighboring countries, e.g. 3.4% in Turkey [23]. Other studies have shown that Iranian people have high rates of high HDL-cholesterol as another criterion of metabolic syndrome. Azizi et al, have shown that in TLGS, 73% of participants had low HDL-cholesterol, which is more than what had been previously reported in many western countries [22].

The other finding that requires further elaboration is the high prevalence of abdominal obesity in Iranian women. While mean WHR in 19 populations studied in WHO MONICA project [36] was from 0.87–0.99 in men and from 0.76–0.84 in women, the mean WHR among men and women in our study was 0.96 in both men and women. The median WHR in Iranian women was 0.95, while the median WHR in a Danish population was 0.80 [37]. Therefore, Iranian women have considerably higher WHR comparing to other countries and as mentioned above, a large proportion of women in our study population had high WHR even with normal BMI. This might be due to genetic predisposition of Iranian women, lower smoking rates, or differences in epigenetic programming of Iranian women.

Prevalence of obesity and metabolic syndrome is increasing in Iran. TLGS showed the trend in rising prevalence of obesity and overweight in Tehran [38]. Considering the constant increase in prevalence of obesity in both US and Iran, Table 2 provides a rough estimate of where our results fall within the trend of obesity in the US. Technological changes that has lowered both the real cost of food and the level of physical activity [39], increased food consumption [40], and decrease in smoking rate [35] are among the reasons suggested for this trend. Also, as mentioned above, the transition toward a Western lifestyle has put a population that is susceptible to obesity–due to factors like epigenetic programming–at high risk of obesity, metabolic syndrome, and their consequences. The fact that women in our study, comparing to American women, showed significantly higher prevalence rates in lower age groups is consistent with the latter hypothesis, as older women have had been less affected by consequences of industrialization.

## Conclusion

Prevalence of obesity and overweight in Iran-as well as other Middle Eastern countries–is as high as the United States. However, Iranian women are more obese than American women, while Iranian men are less obese than their American counterparts. This discrepancy might be due to the low rate of smoking among Iranian women comparing to both Iranian men and American women. Demonstration of this association requires further investigation. Iranian women tend to have higher WHR comparing to many other populations. Further studies are necessary to elucidate the reasons for this finding as well as the necessity of changing the definition of abdominal obesity in Iran based on the distribution of WHR in Iranian population. Prevalence of hypertension and diabetes mellitus in our study was similar to the US population.

## Competing interests

The author(s) declare that they have no competing interests.

## Authors' contributions

HB participated in conception of the research questions, performed statistical analysis and wrote the manuscript. MS participated in coordination of the study, development of database, data management, and helped to draft the manuscript. AP participated in the design and coordination of the study. FK participated in coordination of the study, interpretation of the results, and helped to draft the manuscript. MN and SS participated in coordination of the study. PaulB and PaoloB participated in design of the study and interpretation of results. RM participated in design and coordination of the study, conception of the research questions, interpretation of data and preparation of manuscript. All authors read and approved the final manuscript.

## Pre-publication history

The pre-publication history for this paper can be accessed here:



## Figures and Tables

**Table 1:** Demographic and anthropometric characteristics of the participants in Golestan Cohort Study, Iran.

Demographic/Anthropometric Characteristics		Men (N = 3,787)	Women (N = 5,212)
Age (years)		53.7 (53.4–54.0)	52.2 (52.0 – 52.5)
Rural		41.7%	43.0%
Smoking	Current Use	33.2%	2.2%
	Ever Used	47.0%	2.6%
			
Alcohol Consumption	Current Use	7.2%	0.1%
	Ever Used	17.8%	0.1%
BMI (kg/m^2^)		25.8 (25.6–26.1)	28.6 (28.3–28.8)
BMI Categories	Obesity (BMI = 30 kg/m^2^)	17.0%	36.7%
	Overweight (25 = BMI <30 kg/m^2^)	38.0%	33.1%
	Normal weight (18.5 = BMI<25 kg/m^2^)	40.3%	26.8%
	Underweight (BMI <18.5 kg/m^2^)	4.8%	3.5%
Waist Circumference (cm)		96.0 (95.6–96.4)	98.0(97.3–98.7)
Waist-to-Hip Ratio		0.96 (0.95–0.96)	0.96(0.95–0.97)

**Table 2:** Adjusted* and unadjusted prevalence rates of obesity and overweight in Golestan Cohort Study, Iran, and Third National Health and Nutrition Examination Survey (NHANES III), United States

			Iran	United States	
				
			Golestan Cohort Study	1999–2002	1988–1994
Unadjusted					
	Overweight				
		Overall	63.5 (0.5)^†^	65.2 (0.8)	54.9 (0.8)
		Men	54.9 (0.8)	68.6 (0.9)	59.4 (1.0)
		Women	69.7 (0.6)	62.0 (1.2)	50.7 (1.0)
	Obesity				
		Overall	28.4 (0.4)	30.5 (0.9)	22.3 (0.6)
		Men	17.0 (0.6)	27.5 (0.9)	19.5 (0.7)
		Women	36.6 (0.7)	33.4 (1.2)	25.0 (0.9)
Adjusted					
	Overweight				
		Overall	62.2 (0.5)	65.1 (0.8)	56.0 (0.8)
		Men	53.7 (0.8)	68.8 (0.9)	61.0 (1.0)
		Women	68.6 (0.6)	61.6 (1.2)	51.4 (1.0)
	Obesity				
		Overall	28.0 (0.4)	30.4 (0.9)	22.9 (0.7)
		Men	16.2 (0.6)	27.5 (0.9)	20.2 (0.7)
		Women	34.9 (0.7)	33.2 (1.2)	25.5 (0.9)

*Adjustments for age have been performed in both populations to the 2000 US standard population using 5-year age groups.

† Data presented as: percent (standard error)

**Table 3:** Adjusted* and unadjusted prevalence of hypertension in Golestan Cohort Study, Iran, and Third National Health and Nutrition Examination Survey (NHANES III), United States

		Iran (Golestan Cohort Study)	USA (NHANES III)
**Unadjusted**	Overall	32.5 (0.5) ^†^	30.1 (1.0)
	Men	29.8 (0.7)	27.5 (1.2)
	Women	34.5 (0.7)	32.7 (1.1)
			
**Adjusted**	Overall	33.8 (0.5)	29.9 (0.8)
	Men	29.4 (0.7)	28.7 (1.2)
	Women	35.7 (0.7)	30.5 (0.7)

*Adjustments for age have been performed in both populations to the 2000 US standard population using 5-year age groups.

† Data presented as: percent (standard error)

**Table 4:** Age-adjusted prevalence of obesity and overweight in Golestan Cohort Study (North-East of Iran) and Tehran Lipid and Glucose Study. [21] (Tehran, Iran)

		Golestan Cohort Study	Tehran Lipid and Glucose Study
**Overweight (%)**	Overall	62.2	63.1
	Men	53.7	57.0
	Women	68.6	67.6
			
**Obesity (%)**	Overall	28.0	23.1
	Men	16.2	14.4
	Women	34.9	29.5
